# Supplementary material for: Generation of swine movement network and analysis of efficient mitigation strategies for African swine fever virus
Source: PLoS One. 2019 Dec 5;14(12):e0225785. doi: 10.1371/journal.pone.0225785 (PMC6894757; doi:10.1371/journal.pone.0225785)
Supplement: S1 Appendix — (PDF) [file pone.0225785.s005.pdf]

## Appendix A: Network Generation Algorithms

The basic outline of network generation is described in Algorithm 1.

**input** : $N_F, PIG\_DAT, SF, P, F\_DIST, M\_MIX, K\_DAT, SHP\_DAT$
**output** : $G_F, G_P$

`// Assigns different operation types to individual farms uniformly at random.`

$F\_TYPE \leftarrow F\_TYPE\_GEN(F\_DIST, N_F)$;

`// Generates a directed farm graph with weighted (shipment rate) links.`

$G_F \leftarrow F\_GRAPH\_GEN(M\_MIX, F\_TYPE, N_F, K\_DAT, SHP\_DAT)$;

`// Assigns pigs to different operations based on data obtained from` $NASS$`. Scales the population by a factor` $SF$`.`

$PIG\_LIST \leftarrow PIG\_ALLOT(PIG\_DAT, N_F, SF)$;

`// Generates a pig level graph.`

$G_P \leftarrow P\_GRAPH\_GEN(G_F, N_F, PIG\_LIST, P)$;

**Algorithm 1:** Basic outline of graph generation

In the above box, $N_F$ is the number of farms, $PIG\_DAT$ is the pig distribution data given in Table 4, $SF$ is the scaling factor used to scale the pig level graph in order to make it computationally feasible in our model, $P$ is the probability of within farm contacts between pigs, $F\_DIST$ is the farm type distribution shown in Table 3, $M\_MIX$ is the mixing matrix shown in Table 1, $K\_DAT$ contains the degree centrality data from Table 2, and $SHP\_DAT$ contains the mean and median shipment data. The farm level and pig level graphs are represented by $G_F$ and $G_P$ respectively. We describe

the $F\_GRAPH\_GEN$ function in more detail in Algorithm 2.

**input** : $M\_MIX, F\_TYPE, N_F, K\_DAT, SHP\_DAT$
**output** : $G_F$
`// Allocates in and out-degrees based on production types.`
$DEG\_ALLOC \leftarrow DEG\_GEN(F\_TYPE, K\_DAT)$;
**for** $SRC\_NODE \leftarrow 1$ **to** $N_F$ **do**
    `// Get the allocated (max) out-degree of source node.`
    $MAX\_KOUT = DEG\_ALLOC[SRC\_NODE, OUTDEG]$;
    **for** $k \leftarrow 1$ **to** $MAX\_KOUT$ **do**
        `// Randomly sample the destination type from the mixing matrix distribution.`
        $DST\_TYPE \sim M\_MIX[F\_TYPE[SRC\_NODE]]$;
        `// Find all the nodes of the given type.`
        $DST\_NODES \leftarrow \{n : n \in [1, N_F]$ and $F\_TYPE(n) = DST\_TYPE\}$;
        `// Pick the destination node which has the largest in-degree gap (max in-degree - current in-degree) to cover up.`
        $DST\_NODE \leftarrow n \in DST\_NODES$ that maximizes $(KIN(n, MAX) - KIN(n, CUR))$;
        `// generate shipment data from log-normal distribution with mean and median shipment values given in` $SHP\_DAT$.
        $SHP\_RATE \sim Lognormal(SHP\_DAT)$;
        `// Create the link with generated shipment rate as weight.`
        $G_F \leftarrow G_F + EDGE(SRC\_NODE, DST\_NODE, SHP\_RATE)$;
    **end**
**end**

**Algorithm 2:** $F\_GRAPH\_GEN$

We also describe another key component of the graph generator, $P\_GRAPH\_GEN$

in Algorithm 3, which generates the pig level network.

**input** : $G_F, N_F, PIG\_LIST, P$
**output** : $G_P$
**for** $f \leftarrow 1$ **to** $N_F$ **do**
  `// List of pigs in the farm` $f$.
  $PIG\_NODES \leftarrow PIG\_LIST(f)$;
  `// Generate Erdos-Renyi graph for list of pig nodes with edge probability` $P$.
  $EDGES = ERDOS\_RENYI(PIG\_NODES, P)$;
  `// Add the generated edges to the pig level graph.`
  $G_P \leftarrow G_P + EDGES$;
**end**
`// Normalize the edge weights to be used as probabilities.`
$G_F^{NORM} \leftarrow G_F / MAX(G_F)$;
**for** *each* $(F1, F2)$ *pair where* $F1, F2 \in G_F$ *and* $F1! = F2$ **do**
  `// Probability of movement from farm F1 to any other farms.`
  $P_{FROM} \leftarrow G_F^{NORM}(F1, ALL)$;
  `// Probability of movement from any farm to farm F2.`
  $P_{TO} \leftarrow G_F^{NORM}(ALL, F2)$;
  **for** *each* $P1 \in PIG\_LIST(F1)$ **do**
    **for** *each* $P2 \in PIG\_LIST(F2)$ **do**
      $r \sim Uniform(0, 1)$;
      **if** $r \leq P_{FROM} \times P_{TO}$ **then**
        $G_P \leftarrow G_P + EDGE(P1, P2)$;
    **end**
  **end**
**end**

**Algorithm 3:** $P\_GRAPH\_GEN$
