# Supplementary material for: Generation of swine movement network and analysis of efficient mitigation strategies for African swine fever virus
Source: PLoS One. 2019 Dec 5;14(12):e0225785. doi: 10.1371/journal.pone.0225785 (PMC6894757; doi:10.1371/journal.pone.0225785)
Supplement: Attachment — Submitted filename: Rebuttal Letter.pdf [file pone.0225785.s007.pdf]

Rebuttal Letter

We would like to thank the editor for his useful suggestions. We have addressed all the suggestions in our current version and we believe the manuscript has been improved. In this letter, our responses are written in purple. We have included a marked-up copy (pdf) of the manuscript where changes are highlighted in yellow.

Could you please make some small edits:
line 52, a a......

Thank you for identifying the problem. We have fixed it.

Betwenness--> Betweenness

Thank you. We have corrected it.

Delete all double spaces and replace by singles ones.

Thank you for the suggestion. We searched for double spaces between words in the latex file. We found a few and fixed them.

Standardize the use of capitals in titles of all articles in the reference list (use lowercase, see Plos One guidelines)

Thank you for the suggestion. We have fixed the unnecessary uppercases in the references. Please check the marked-up manuscript.
